# Brain structure in triple X syndrome: regional gray matter volume and cortical thickness in adult women with 47,XXX karyotype

**DOI:** 10.1186/s11689-025-09608-6

**Published:** 2025-04-01

**Authors:** Gregor Domes, Marie-Anne Croyé, Petra Freilinger, Andreas Bohlscheid, Winfried A. Willinek, Jobst Meyer

**Affiliations:** 1 Department of Biological and Clinical Psychology, University of Trier, Johanniterufer 15, 54290 Trier, Germany; 2 Institute for Cognitive and Affective Neuroscience, University of Trier, Trier, Germany; 3 Department of Neurobehavioral Genetics, University of Trier, Trier, Germany; 4 Genetikum, Neu-Ulm, Germany; 5 Department of Radiology, Neuroradiology, Sonography and Nuclear Medicine, Hospital of the Barmherzige Brüder Trier, Trier, Germany

**Keywords:** 47,XXX, Triple X syndrome, Trisomy X, Magnetic resonance imaging, Morphometry, Amygdala, Hippocampus, Basal ganglia, Cerebellum

## Abstract

**Background:**

Changes in the brain structure of women with Triple X syndrome (karyotype 47,XXX) have been described in a few studies to date, including reduced total brain volume and regional reductions in gray substance in cortical and subcortical areas. However, the empirical evidence from adults is very limited and group comparison on a voxel-wise basis for gray matter volume and cortical thickness is still missing.

**Methods:**

Using voxel-based morphometry (VBM) and surface-based morphometry (SBM), we investigated regional gray matter changes in a sample of *n* = 20 adult women (aged 18–49 years) with 47,XXX karyotype using T1-weighted 3T MRI scans.

**Results:**

Compared to an age- and education-matched control group (and controlled for differences in total intracranial volume), the VBM revealed decreased regional gray matter volumes in the hippocampus, amygdala, parts of the basal ganglia, insula, prefrontal areas and cerebellum. To a lesser extent, we also noted specific reductions in cortical thickness in a smaller part of those regions.

**Conclusion:**

The observed network is significantly involved in the processing of cognitive, affective, and social stimuli and might be a potential neuronal correlate of the autism-like social-cognitive problems described in 47,XXX in the literature.

**Supplementary Information:**

The online version contains supplementary material available at 10.1186/s11689-025-09608-6.

## Background

Triple X syndrome (karyotype 47,XXX), also known as trisomy X, is < a sex chromosomal aneuploidy characterized by the presence of an additional X chromosome in females [1]. It has an estimated prevalence of about 0.1% in female newborns [2]. However, only few cases are clinically diagnosed, possibly due to the inconspicuous phenotype and lack of clinical symptoms [3, 4].

Research has identified impairment of different domains comprising motor development, speech and language, educational attainment, socio-economic status and interpersonal relationships in children and adolescents with 47,XXX [5–8]. Notably, elevated rates of neurodevelopmental conditions like autism spectrum disorder (ASD) and attention deficit hyperactivity disorder (ADHD), as well as associated socio-cognitive and behavioral problems have been reported [8–13]. Individuals with 47,XXX also exhibit heightened susceptibility to (social) anxiety, depression, and psychotic disorders compared to the general population [13–15].

In the cognitive domain, mild learning difficulties and executive function deficits are prevalent among those with 47,XXX during childhood and adolescence [16, 17]. Notably, their mean full-scale IQ (FSIQ) tends to be lower, with a leftward shift in the IQ normal curve compared to 46,XX controls, with verbal IQ (VIQ) often more affected [7]. Furthermore, impairments in everyday social functioning, social cognition, and behavior have been observed in both pediatric and adult samples with 47,XXX [18–21].

A few neuroimaging studies have demonstrated the presence of structural brain abnormalities in individuals with 47,XXX, including alterations in total brain and subcortical volumes, cortical thickness and surface area, and cortical folding in children and adolescents [22–26]. Note that these studies (except for Lenroot et al., 2014) relied on a largely identical group of youth with a mean age of 12 years. In sum, those analyses indicate reduced total brain volume (TBV), and reductions in amygdala and hippocampus gray matter, which for the amygdala was still significant after controlling for reduced TBV [24]. Consistently, in adults, an early study with 10 women revealed initial evidence for smaller amygdala volumes in 47,XXX, although this effect was not significant due to limited statistical power [27]. Beyond these preliminary findings, there is only one study focusing on alterations in cortical, subcortical, and lateral ventricle volumes in an adult sample (Serrarens et al., 2022). Using T1 images acquired with an ultra-high field 7 T scanner, Serrarens and colleagues reported reduced gray matter volumes of the basal ganglia (caudate nucleus, putamen, pallidum, nucleus accumbens), thalamus, and hippocampus, presumably associated with enlarged lateral ventricles. In addition, reduced gray matter was detected in the superior frontal gyrus and superior temporal gyrus in 47,XXX participants compared to controls. Although cortical thickness was not specifically altered in 47,XXX, there was a negative correlation between emotion recognition performance and cortical thickness in the left caudal anterior cingulate cortex and positive with cortical thickness in left lateral occipital, left pericalcarine, and right superior parietal cortex in 47,XXX [28]. In sum, the empirical evidence is currently very limited and heterogeneous (see supplementary Table S1), Hence it is hard to draw a consistent picture based on these very few studies. To put it cautiously, the most consistent finding seems to be reduced total brain volume and reduced gray matter and white matter in subcortical (including amygdala, hippocampus and basal ganglia) and some cortical areas. Initial results also suggest that within group variance in subcortical and cortical structures is related to social functioning.

With the present study, we aimed at replicating reported results in an independent adult sample of women with 47,XXX compared to an age- and education-matched control group. In extending the earlier studies, we took a whole brain voxel-based morphometry (VBM) approach to more precisely localize gray volumetric alterations in 47,XXX and used surface-based morphometry (SBM) to discern potential local alterations in cortical thickness. Finally, we explored potential associations with self-reported traits related to social cognition and psychopathology. Based on the evidence presented above both related to the previous structural imaging studies and phenomenological similarities with ASD, we expected to observe GM volume reductions and reduced cortisol thickness in amygdala, hippocampus, putamen, pallidum, caudate, and thalamus, as well as decreased total brain volume and enlarged ventricles. We exploratively tested associations between regional brain volumes and different self-report measures in the social-cognitive domain such as empathy, autistic traits, as well as symptoms of depression and anxiety.

## Methods

### Participants

Women with a 47,XXX karyotype were recruited via a German Triplo-X support group, specialists in human genetics, and social media. They were included on the basis of a confirmed 47,XXX karyotype. Controls matched for sex, age and education (highest school-leaving qualification, as detailed in Table S2 provided in the supplementary materials) were recruited via flyers, digital newsletters and local advertising. Participants in both groups were only included if they were 18 years of age or older and spoke the German language at native speaker level. Participants with a history of traumatic brain injury, neurological damage, current intake of psychoactive drugs, magnetic implants in their body, claustrophobia, or pregnancy were excluded from the study. Initially, 54 women diagnosed with 47,XXX karyotype and 64 controls expressed interest in participating. After applying inclusion and exclusion criteria, our final analysis included data from 20 women with 47,XXX karyotype (aged 18–49 years) and 23 controls (aged 18–52 years). The recruitment took place between 2021 and 2023.

The study was conducted in accordance with the Declaration of Helsinki. The protocol was approved by the ethics committee of the state medical association Rhineland-Palatinate (#2022–16572). Participants gave written-informed consent before participation. All participants were compensated for their participation and any travel expenses were reimbursed.

### Psychological assessment

General intellectual abilities (IQ) were measured with the Culture Fair Test 20 (CFT-20) revised version. The CFT 20-R measures the general intellectual level (basic intelligence) in terms of Cattell’s “General Fluid Ability”. We used part 1 of the German CFT-20-R [29] containing 56 items in four subtests (series continuation, classifications, matrices and topological reasoning). Test-retest reliability for the subtest is r_tt_ = 0.80 and the internal consistency (Cronbach’s alpha) is α = 0.94. Raw scores were transformed based on the IQ norms provided in the 2nd edition.

Verbal intelligence was estimated via a vocabulary test (WST) [30] designed to measure verbal abilities. The WST consists of 40-word recognition items, each containing one target word and five distractors. The split-half reliability (Spearman-Brown) is *r* =.95, the internal consistency is α = 0.94. Raw scores were transformed based on the IQ norms provided.

A short version of the Autism Quotient, the AQ-k [31] was used to assess traits related to the autism spectrum. The German short version of the original AQ [32] comprises 33 items rated on 4-point Likert scales and analyzed in dichotomized form, resulting in total scores between 0 and 33 points. The AQ-k has acceptable reliability (internal consistency for the subscales: α = 0.65 − 0.87 and for the total score α = 0.79). In addition, the AQ-k has been shown to distinguish between individuals on the autism spectrum and typically developing individuals with a high sensitivity and specificity at a cutoff score of 17 points [31].

The Interpersonal Reactivity Index (IRI) [33] contains 28 items and measures four traits related to empathy: perspective taking (the tendency to adopt others’ viewpoints), fantasy (the tendency to identify with fictional characters in books, movies, and plays), empathic concern (feelings of compassion and concern for others), and personal distress (feelings of discomfort and anxiety in response to others’ distress). For the present analysis, we calculated total scores summing up the scales perspective taking, fantasy, and empathic concern [33]. The internal consistency for the subscales is acceptable and varies between α = 0.68 − 0.79.

Levels of trait anxiety were assessed with the trait scale of the State-Trait Anxiety Inventory (STAI-T) [34]. The trait scale of the STAI assesses individual differences in the tendency to experience and show fear reactions based on 20 items (4-point Likert-scale). The internal consistency is α = 0.90 and the test-retest reliability has been estimated at r_tt_ = 0.67 − 0.90.

The Beck-Depression Inventory (BDI) was used to assess depressive symptoms [35]. Containing 21 items, the BDI assesses the severity of a depression based on the clinical symptoms according to DSM and ICD. The internal consistency has been estimated at approx. α = 0.90 in different samples and the test-retest reliability r_tt_ = 0.78 in a community sample.

### Magnetic resonance imaging

T1-weighted images were acquired on a 3T Philips Ingenia Elition X equipped with a 15-channel head coil. Sagittal images covering the whole head were acquired with an 3D MPRAGE sequence with the following parameters: 208 sagittal slices (256 × 256 in-plane resolution), 1 mm isotropic voxels, flip angle = 8°, TR = 5.22 ms, TE = 2.36 ms. Total scan duration was approx. 6:30 min.

### Morphometric analysis

We applied four different analyses pipelines to the structural images: (a) Voxel-based morphometry (VBM) analysis focusing on voxel-wise gray matter volume differences, (b) a respective region-based analysis focusing on regional gray matter volume (i.e., a voxel-based ROI analysis), (c) a surface-based morphometry (SBM) analysis focusing on point-wise cortical thickness, and (d) a corresponding region-based analysis focusing on regional cortical thickness (i.e., a surface-based ROI analysis).

#### Processing of images

For further analyses, T1 images were spatially preprocessed and segmented with the Computational Anatomic Toolbox (CAT12.9, 2550;, 36) implemented in Statistic Parametric Mapping (SPM12, 7771; https://www.fil.ion.ucl.ac.uk/spm/software/spm12/) running on Matlab (R2018a). In a first step, T1 images were normalized and segmented into gray matter, white matter, and CSF by using the probability maps as priors provided within CAT12. As a result of segmentation, bias, noise and global intensity corrected, modulated and normalized gray and white matter images were saved for regional volumetric analysis. For quality control, we calculated deviations in individual gray matter image intensities from the global mean. Those images deviating from the mean with > 1.5 IQR were visually inspected for artefacts (*n* = 3). None of the scans had to be excluded, as all scans were of good data quality. Images were then smoothed with a 3D Gaussian kernel of 6 mm FWHM for VBM, and with a kernel of 20 mm FWHM for SBM. We also calculated individual total intracranial volumes (TIV) for group comparison and to be included as a covariate in the general linear model for group comparison of regional gray matter volumes, in both VBM and ROI-based analyses.

#### Voxel-based morphometry

For group comparison of VBM regional gray matter volume, we set up a GLM with one factor (group) and age and TIV as covariates. After estimation, two contrasts were defined to test for regional gray matter volume decrease vs. increase in the 47,XXX group. The resulting t-maps were written with at a peak-voxel threshold of *p* < .05 with a family-wise error (FWE) correction based on random field theory [37]. A cluster extent threshold of k > 100 was also applied. Automatic anatomical labelling (AAL3v1) was used to label peak voxels in cortical and subcortical regions [38]. The VBM outcomes were overlaid onto coronal sections of MNI152 average template using MRIcroGL (v1.2; 39).

#### Voxel-based ROI-analysis

The voxel-based ROI analysis relied on Neuromorphometrics atlas (http://neuromorphometrics.com/) to calculate regional gray matter, white matter and ventricular volumes. The labeling based on the Neuromorphometric atlas implemented in CAT12 differentiates and labels 136 regions including ventricles, vessels, and total volumes of the CSF, cerebrum, and cerebellum. Results on gray matter volumes were tested for significance with an FDR-corrected threshold of *p* < .05 and projected on the Freesurfer average template (https://surfer.nmr.mgh.harvard.edu/) within CAT12.

#### Surface-based morphometry

Surface data to estimate regional cortical thickness were written during segmentation within CAT12, see above. Similar to the VBM analysis, we set up a GLM with group as the only factor, but without controlling for TIV. After estimation, again two contrasts were defined to test for group differences in regional cortical thickness. The resulting t-maps were thresholded at *p* < .05 (FWE-corrected) and at an exploratory threshold of *p* < .001 (uncorrected).

#### Surface-based ROI analysis

The surface-based ROI analysis employed the DK40 atlas [40] to calculate regional cortical thickness for predefined ROIs. Those results were projected on the Freesurfer average template (https://surfer.nmr.mgh.harvard.edu/) with a Holm-Bonferroni corrected threshold of *p* < .05.

#### Correlation analysis

To explore potential associations between regional GM volume and autistic and empathic traits on the one hand and anxiety/depressive symptoms on the other hand, we calculated Pearson’s correlation coefficients between AQ, IRI, BDI and STAI scores with regional GM volumes for bilateral amygdala, hippocampus, putamen, and cerebellum based in the voxel-based ROI analysis described above. Correlation coefficients were calculated for the two study groups separately.

## Results

### Study group characteristics

Study groups did not differ significantly in age, education, general and verbal IQ, empathy, trait anxiety or depression (all *p* > .10) but 47,XXX exhibited more pronounced autistic traits (*p* < .05; Cohen’s d = 0.82) - see Table 1.

**Table 1:** Demographic and psychometric characteristics

	47,XXX (*n* = 20)			Controls (*n* = 23)					Statistical Test			
	M	SD			M	SD				t	p	d
Age	27.4	9.2			28.7	9.5				-0.46	0.650	-0.14
Education^1^	10.6	3.1			11.1	1.7				-0.71	0.480	-0.22
General IQ (CFT-20)	98.8	16.9			103.7	11.8				-1.10	0.276	-0.34
Verbal IQ (WST)	96.2	17.3			97.4	13.4				-0.27	0.792	-0.08
Autistic traits (AQ-k)	14.9	5.7			9.9	6.4				2.68	0.010	0.82
Empathy (IRI)	54.1	12.7			54.7	14.2				-0.17	0.869	-0.05
Anxiety (STAI-T)	47.8	10.8			44.4	10.9				1.01	0.316	0.31
Depression (BDI)	16.6	9.9			12.6	8.6				1.32	0.193	0.41

Notes. ^1^ Years in school; AQ-k: Autism Quotient – short version; BDI: Beck Depression Inventory; CFT-20: Culture Fair Test 20; IRI: Interpersonal Reactivity Index; STAI-T: State-Trait Anxiety Inventory – Trait Scale; WST: Vocabulary Test (“Wortschatztest”)

### Voxel-based morphometry

#### Total intracranial volume and ventricles

TIV was significantly lower in 47,XXX women (m = 1317, SD = 85 cm^3^) than in controls (m = 1403, SD = 75 cm^3^), t(41)= 3.505, *p* < .001. In addition, the group difference in total ventricle volume approached significance after controlling for TIV (F[1,40] = 3.95, *p* =.054), with 47,XXX women showing slightly enlarged ventricle volumes (m = 15.3, SD = 6.5 cm^3^) than controls (m = 13.4, SD = 4.4 cm^3^).

#### Gray matter volume

**Fig. 1:**
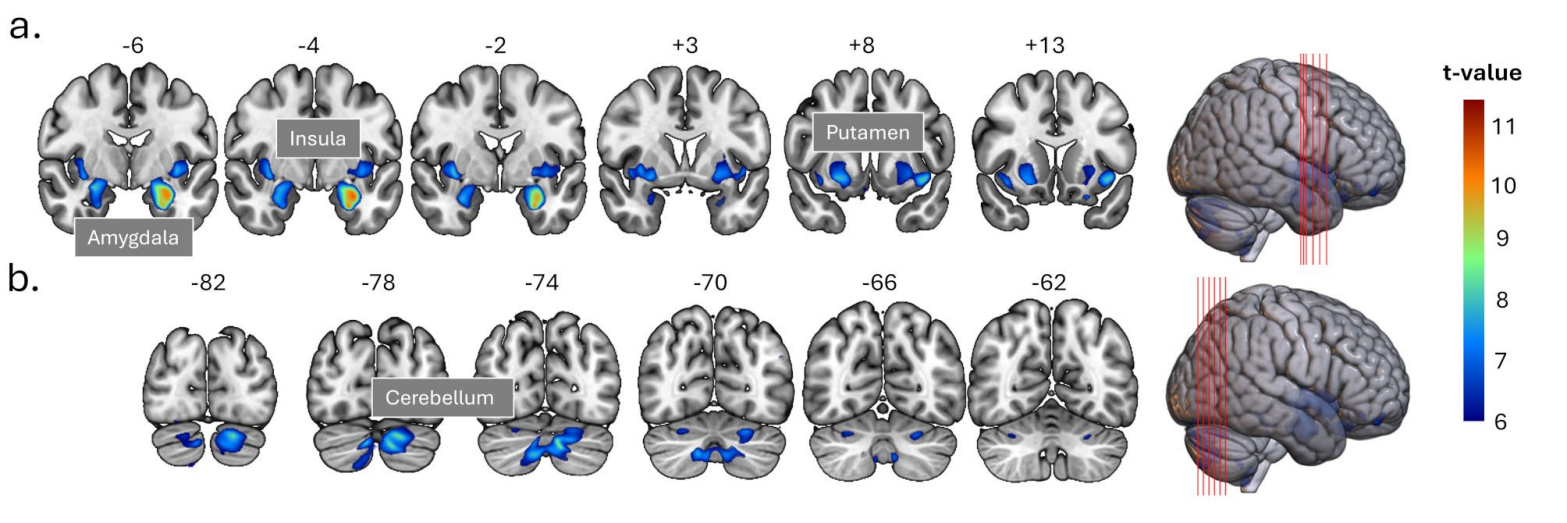
Results of voxel-based morphometry (VBM). Statistical parametric maps (SPM) are projected on coronal slices of the MNI152 template illustrating regions with reduced GM volume in women with a 47,XXX karyotype. (**a**) Extended clusters showing gray matter volume reduction comprising hippocampus, amygdala, putamen, insula. (**b**) Extended clusters in the cerebellum. Results were FWE-corrected with a voxel threshold of T > 6.14; *p* < .05

For the contrast (controls > 47,XXX) representing decreased GM volume in women with 47,XXX, we found significant GM volume reductions in four extended clusters comprising [1] left hippocampus, amygdala, and insula (k = 3851; x, y,z=-29, -6, -20; T = 11.29; *p* < .000001) [2], the bilateral cerebellum (k = 4991; x, y,z = 14, -72, -41; T = 10.43 *p* < .000001) [3], right amygdala (k = 892; x, y,z = 27, -6, -15;T = 9.48; *p* < .00001) [4], right putamen and insula (k = 1680; x, y,z,=35, 0, -3; T = 8.73; *p* < .0001) - see the first four clusters in Table 2; Fig. 1.

In addition, we identified smaller, scattered clusters in the cerebellum, inferior frontal cortex, mainly in the orbitofrontal cortex, and the fusiform gyrus (see Table 2 lower part). Notably, in the reverse contrast testing for increased GM volume in the 47,XXX group compared to controls, none of the voxels were significant.

**Table 2:** VBM GLM results for the contrast controls > 47,xxx. Significant clusters with decreased GM volume in 47,XXX at a voxel threshold of *p* < .05 (FWE-corr.) and a cluster size threshold of k > 100. A complete list of significant clusters can be found in supplementary table S3

Region^1^	Size		MNI coordinates				Peak voxel		
	mm^3^		x	y	Z		T	Z	p
Hippocampus L	3851		-29	-6	-20		11.29	7.43	0.00000007
Amygdala L			-26	-3	-30		10.72	7.23	0.00000033
Insula L			-41	11	-9		8.70	6.41	0.00005541
Cerebellum 8 R	4991		14	-72	-41		10.43	7.12	0.00000070
Cerebellum Crus2 R			9	-78	-35		9.40	6.72	0.00000917
Cerebellum Crus2 L			-8	-75	-33		9.35	6.70	0.00001023
Amygdala R	892		27	-6	-15		9.48	6.75	0.00000741
Amygdala R			29	-3	-24		8.85	6.48	0.00003713
Putamen R	1680		35	0	-3		8.73	6.43	0.00005028
Putamen R			24	6	-8		8.25	6.21	0.00017707
Insula R			38	17	-11		7.59	5.89	0.00102743
Cerebellum 4 5 L	112		-15	-33	-20		8.41	6.28	0.00011584
Cerebellum 4 5 L			-11	-38	-14		7.34	5.76	0.00200160
Cerebellum 9 L	112		-14	-44	-54		7.81	5.99	0.00057612
Cerebellum 9 L			-15	-50	-47		6.27	5.17	0.03485522
OFC ant R	109		30	48	-20		7.75	5.96	0.00067992
Cerebellum 9 R	299		5	-48	-65		7.65	5.92	0.00087472
Cerebellum 9 R			9	-53	-48		7.39	5.78	0.00174805
Cerebellum 9 R			3	-54	-71		7.09	5.62	0.00399479
OFC post L	235		-23	14	-23		7.57	5.87	0.00110046
OFC post L			-20	23	-20		7.43	5.80	0.00158340
OFC med L			-15	20	-27		7.01	5.58	0.00491704
OFC post R	192		26	27	-14		7.44	5.81	0.00154603
OFC med R			18	23	-20		7.16	5.66	0.00327685
OFC med R			17	23	-29		7.08	5.62	0.00402730

Notes. ^1^ Peaks are given for cluster peak voxels and local maxima within the clusters

**Fig. 2:**
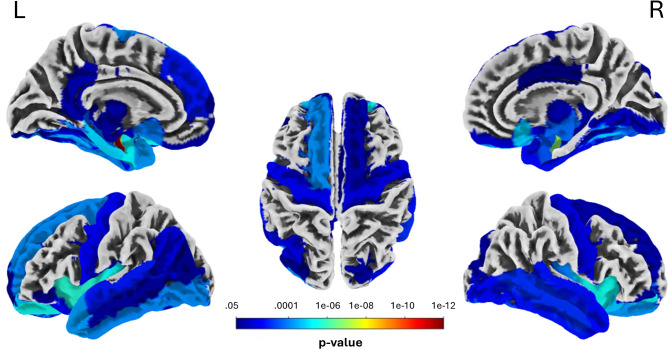
ROI-based analysis showing significant ROI of decreased gray matter volume in women with 47,XXX compared to controls. ROIs were defined using the Neuromorphometrics atlas. Results were FDR-corrected at *p* < .05

In addition to the voxel-wise analysis, region-of-interest (ROI)-based analysis revealed corresponding results: We identified significant ROIs with reduced gray matter volume in (all cluster *p* < .05 Holm-Bonferroni-correction): Amygdala, Hippocampus, Insula, Putamen, Thalamus, orbitofrontal cortex, fusiform gyrus, parts of the temporal lobe, frontal cortex – see Fig. 2; Table 3. Robust effects were observed in limbic areas (Cohen’s d > 1.35), especially in the bilateral amygdala (Cohen’s d = 2.38 and d = 3.51, respectively). Again, reverse comparison testing for increased regional gray matter volume in the 47,XXX group revealed no significant ROI. None of the regional gray matter volumes in amygdala, hippocampus and putamen were associated with lateral ventricle volume (all correlations with *p* > .05).

**Table 3:** Results of ROI-analysis for the contrast controls > 47,xxx. Significant rois with D.creased GM volume in 47,XXX (all *p* < .05; Holm-Bonferroni-corr.). Effect sizes are reported as Cohen’s D

	Left hemisphere					Right hemisphere			
Region	T	p	d		Region	T	p	d	
*Limbic*									
Amygdala	11.47	0.000001	3.51		Amygdala	7.78	0.000001	2.38	
Hippocampus	5.38	0.000189	1.64		Hippocampus	4.41	0.004718	1.35	
Parahippocampal g.	6.11	0.000012	1.87						
Entorhinal cortex	6.58	0.000005	2.01		Entorhinal cortex	5.25	0.000273	1.61	
Anterior insula	6.69	0.000002	2.05		Anterior insula	6.62	0.000003	2.02	
Posterior insula	6.39	0.000004	1.95		Posterior insula	5.28	0.000103	1.61	
*Subcortical*									
Putamen	6.59	0.000005	2.01		Putamen	5.75	0.000036	1.76	
					Ncl. Accumbens	4.56	0.003110	1.39	
Ventral DC	3.93	0.019503	1.20		Ventral DC	5.48	0.000158	1.68	
Thalamus proper	3.17	0.029940	0.97		Thalamus Proper	4.00	0.010376	1.22	
Cerebellum ext.	5.76	0.000020	1.76		Cerebellum ext.	6.23	0.000012	1.90	
*Frontal lobe*									
Medial orbital	6.63	0.000003	2.03		Medial orbital	5.75	0.000047	1.76	
Anterior orbital	5.27	0.000014	1.61		Anterior orbital	6.16	0.000003	1.88	
Posterior orbital	4.45	0.004015	1.36		Posterior orbital	4.90	0.000018	1.50	
					Lateral orbital g.	3.58	0.023680	1.09	
Superior frontal	4.74	0.000060	1.45		Superior frontal	4.02	0.011879	1.23	
Sup. frontal medial	4.74	0.001381	1.45						
Inferior frontal g.	3.56	0.030884	1.09						
Frontal pole	3.56	0.037372	1.09						
G. rectus	3.41	0.018745	1.04		G. rectus	3.41	0.005490	1.04	
Basal forebrain	4.01	0.011522	1.23		Basal forebrain	4.32	0.005520	1.32	
Subcallosal area	5.13	0.000096	1.57		Subcallosal area	5.65	0.000014	1.73	
					Middle cingulate	3.44	0.049012	1.05	
Posterior cingulate	4.09	0.012901	1.25						
Precentral	4.50	0.002689	1.38		Precentral	4.06	0.003738	1.24	
Central operculum	3.55	0.014029	1.09						
					Frontal operculum	2.91	0.017958	0.89	
*Temporal lobe*									
Occipital fusiform	3.70	0.035047	1.13		Occipital fusiform	5.26	0.000255	1.61	
Fusiform	4.20	0.003025	1.28		Fusiform	4.12	0.007791	1.26	
Inferior temporal	5.30	0.000065	1.62		Inferior temporal	4.78	0.001426	1.46	
Superior temporal	4.08	0.001235	1.25		Superior temporal	4.54	0.001499	1.39	
Temporal pole	4.73	0.000046	1.45		Temporal pole	4.26	0.001859	1.30	
Planum polare	4.00	0.008862	1.22						
Middle temporal	3.79	0.021667	1.16		Middle temporal	4.38	0.000675	1.34	
					Lingual	4.24	0.007276	1.30	
Angular	4.36	0.004887	1.33						
*Occipital lobe*									
Inferior occipital	5.71	0.000070	1.75		Inferior occipital	4.13	0.001732	1.26	
Middle occipital	3.85	0.026919	1.18						
					Superior occipital	2.12	0.020396	0.65	

### Surface-based morphometry

#### Cortical thickness

**Fig. 3:**
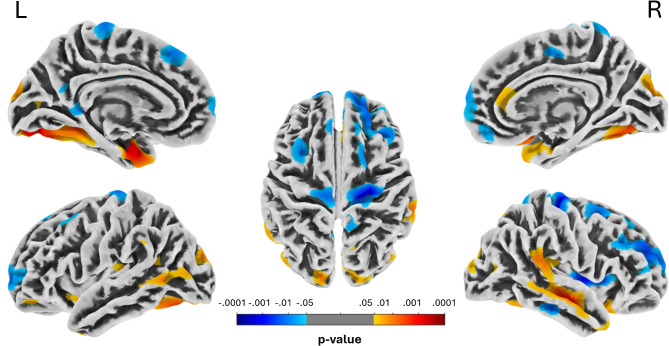
Point-wise cortical thickness analysis showing significant clusters of altered cortical thickness in women with 47,XXX compared to controls. Blue clusters indicate increased vs. red areas indicate reduced cortical thickness in 47,XXX. Results are presented uncorrected at *p* < .05 for illustrative purpose

The contrast controls > 47.XXX for point estimate of cortical thickness revealed no significant voxels at *p* < .05 (FWE-corr.). At a more liberal, explorative level of *p* < .001 (uncorr.), we detected three clusters of reduced cortical thickness in 47,XXX: left visual cortex (BA 17/18, k = 96; x, y,z=-16,-83,-14; T = 4.19; Z = 3.79; *p* =.000074), entorhinal cortex (BA 28; k = 46; x, y,z=-27,-4,-27; T = 4.01; Z = 3.65; *p* =.00014), right inferior frontal gyrus (BA 44/45; k = 49; x, y,z = 49,-11,-16; T = 3.59; Z = 3.32; *p* =.00045) – red clusters in Fig. 3. The reverse contrast of increased cortical thickness in 47,XXX again revealed no significant cluster at *p* < .05 (FWE-corr.), but three clusters at *p* < .001 (uncorrected): supplementary motor area (BA 6; k = 49; x, y,z = 26,-21,69; T = 4.13; Z = 3.75; *p* =.00009), right inferior frontal gyrus (BA 44/45; k = 45; x, y,z = 39,1,4; T = 3.75; Z = 3.45; *p* =.00028) and right mid temporal gyrus (BA 21; k = 15; x, y,z = 54,-30,-20; T = 3.74; Z = 3.44; *p* =.00029)– blue clusters in Fig. 3.

**Fig. 4:**
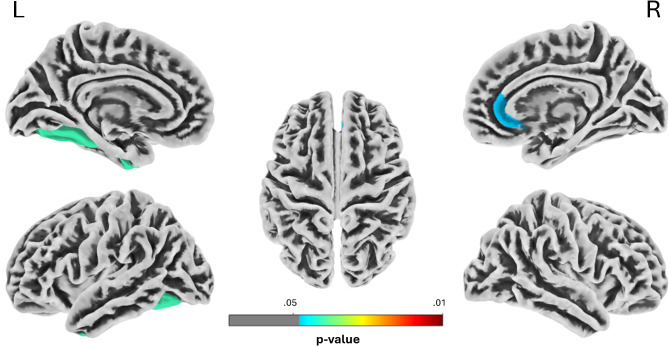
ROI-based thickness analysis showing significant ROI of decreased cortical thickness in women with 47,XXX compared to controls. ROIs were defined based on the DK40 atlas. The results were Holm-Bonferroni corrected and thresholded at *p* < .05

The ROI-based analysis revealed two significant ROIs (Holm-Bonferroni corrected) of decreased cortical thickness in 47,XXX women: the left fusiform gyrus (T = 2.79; Z = 2.65; *p* =.0358; Cohen’s d = 0.85) and the right rostral anterior cingulate cortex (T = 1.72; Z = 1.76; *p* =.0470; Cohen’s d = 0.53) – see Fig. 4.

### Associations between brain morphology and psychometric characteristics

Explorative correlation analyses revealed significant correlations between bilateral amygdala/hippocampus GM volumes and self-reported empathy (IRI), anxiety (STAI-T) and depression (BDI) in women with 47,XXX (see Table 4). Higher regional GM volumes in amygdala and hippocampus was associated with higher levels of self-reported empathy, and lower levels of anxiety and depression. In controls, none of these correlations were significant (all *r* <.40; all *p* > .05). After controlling for age and TIV, only the correlations between the left amygdala and STAI, right amygdala and STAI and BDI, and right hippocampus and STAI remained significant. A complete partial correlation matrix (controlled for age and TIV) for both groups is included in the supplementary materials, see Table S4.

**Table 4:** Correlations between autistic traits (AQ), empathy (IRI), anxiety (STAI-T), and depression (BDI) and regional GM volume in selected rois in women with 47,XXX; asterisks indicate significant Pearson’s correlations (uncorrected)

	AQ	IRI	STAI	BDI
Amygdala L	− 0.11	0.54*	− 0.58**	− 0.60**
Amygdala R	− 0.09	0.55*	− 0.56*	− 0.53*
Hippocampus L	− 0.17	0.47*	− 0.55*	− 0.52*
Hippocampus R	− 0.15	0.41	− 0.50*	− 0.53*
Putamen L	− 0.01	0.33	− 0.15	− 0.11
Putamen R	− 0.04	0.36	− 0.13	− 0.13
Cerebellum L	− 0.12	0.37	− 0.38	− 0.25
Cerebellum R	− 0.17	0.39	− 0.33	− 0.24

## Discussion

Controlled studies investigating brain structure in girls with a 47,XXX karyotype are scarce, and even fewer in adults. With the present study, we compared a group of 20 adult women with 47,XXX with an age- and education-matched control group regarding regional gray matter volume and regional cortical thickness. VBM analysis revealed marked reductions of gray matter volume in the inferior and medial temporal lobe including the hippocampus, amygdala, and fusiform regions, medial frontal and orbital regions, bilateral insula, basal ganglia including putamen and caudate, and several clusters in the cerebellum. SBM analysis revealed cortical thickness reduction in some of these regions, however these clusters were less extended, and the effects were considerably smaller in magnitude compared to the VBM results. Overall, these regional effects were robust after controlling for TIV, which was reduced in the 47,XXX group. As expected, the 47,XXX group exhibited lower TIV, slightly enlarged ventricles, higher levels of self-reported autistic traits, but comparable intellectual abilities and normal levels of self-reported anxiety and depression. Exploratively, we observed that amygdala/hippocampus GM volume was a positive predictor for self-reported empathy and a negative predictor for anxiety and depression in women with 47,XXX. Although significant, albeit moderate, we observed correlations specifically in the 47,XXX group which should be interpreted very cautiously as structural neural alterations could predispose to social-cognitive problems. Further conclusions would require large-scale correlative studies, ideally with a longitudinal design.

Reduced gray matter volume in the basal ganglia has been reported in girls with 47,XXX [26] and in adult women [28]. While these studies reported volume reductions in different parts of the basal ganglia (predominantly in pallidum and caudate), we observed decreased regional gray matter volume in the right putamen and caudate. Given that these regions are involved in a wide range of cognitive, affective and motor processes, the potential functional impact of the structural alterations we observed remains speculative. However, given the reported impairments in cognition, affect, and motor skills in 47,XXX (e.g. 3, 7, 9, 14, 18) and the fact that structural anomalies have been reported in the basal ganglia in ASD [41, 42], it seems plausible that these structural changes could also play a role in the development of the social cognitive impairments described for 47,XXX.

The first remarkable finding of the present study is the strong effect detected in the bilateral medial temporal lobe, including volume reduction in hippocampus, amygdala, and parahippocampal gyrus. Reduced amygdala volume in 47,XXX has been reported in girls [24] and, as a trend, also in adult women [27]. However, a recent study failed to confirm those results in adults [28]. Interpreting these contradictory findings is complicated by the fact that very different methodologies were applied to obtain the volumetric data in small subject samples. Patwardhan and colleagues manually segmented the amygdala and hippocampus, while Nadig et al. and Serrasens et al. used different automated procedures to determine ROIs. Although manual segmentation by trained raters is in principle a precise and reliable method, the statistical power (*n* = 10) in the study by Patwardhan et al. (2002) [27] was limited. In addition, since 1.5 Tesla T1-weighted scans were used, the contrast may have been limited when segmenting the amygdala and hippocampus. In the study by Serrarens et al. (2022) [28], segmentation was performed with Freesurfer and volumes were estimated for predefined ROIs. While in this study sample size was larger and the T1 scans used were presumably of higher quality (scanned at 7 Tesla), the ROI-only approach may be less sensitive at detecting effects in the amygdala. In our study, taking a voxel-based approach and applying advanced segmentation algorithms in CAT12, we were able to detect decreased regional volumes in the hippocampus and amygdala, both within an extensive cluster with local peaks in the hippocampus and amygdala. It seems possible that the diverse gray matter segmentation and estimation algorithms applied in different studies may explain subtle differences in the amygdala. However, the effects may simply have been larger in our study sample.

As part of a larger network (the so-called “social brain”), the amygdala plays an essential role in the representation of emotional processes and is of central importance for social-cognitive functions [43–45]. We chose to focus on autism in this discussion due to the previously reported autism-like features in 47,XXX [9, 10, 13], and the fact that in our study women with 47,XXX reported higher levels of autistic traits. Accordingly, recent reviews have come to the conclusion that structural alterations and functional impairments in the amygdala and adjected areas could be the basis of socio-cognitive deficits within the autism spectrum (e.g. 46). Indeed, recent meta-analytic evidence supports the hypothesis that gray matter volume reductions in the amygdala are associated with autism [47]. In this respect, the regionally reduced gray-matter volume in these core regions within the social brain network detected in our sample could also be a pathophysiological factor related to the reported social-cognitive deficits and social difficulties [19, 28, 48] and autism-like features [9, 10] in adult women with 47,XXX. It should be noted that amygdala volume reductions have also been reported in association with other psychiatric conditions, including emotional instability [49], schizophrenia [50], and depression [51], and might therefore be a relatively unspecific phenomenon. However, changes in amygdala structure are also associated with socio-affective problems in these psychiatric disorders, although the exact relationship is not yet well understood.

The amygdala and the hippocampus are parts of the neural circuits underlying cognitive and affective aspects of empathy [52, 53] particularly in processing emotional stimuli, attributing mental states to others, and emotional memory [54]. Both cognitive and affective facets of empathy are affected in autism spectrum disorder [55, 56]. Reduced amygdala volume may contribute to difficulties in recognizing and responding to others’ emotions [54, 57], while grey matter volume reductions the hippocampus might affect the contextualization of social information [58]. In our sample, the positive correlation between amygdala/hippocampus GM volume and self-reported empathy suggests that structural integrity in these regions may play a role in preserving social-cognitive abilities, even within the context of 47,XXX.

The second remarkable finding of this study is the reduced regional volume in the cerebellum in women with 47,XXX karyotype. We are unaware of any other study showing this structural change in individuals with an extra X chromosome. The cerebellum’s regional volume reduction may have implications for movement impairments and tremor. Tremor, in its different forms, has been described in conjunction with 90 chromosome aneuploidies involving sex chromosomes such as Klinefelter syndrome (47,XXY) [59] and Jacobs syndrome (47, XYY) [60]. However, tremor in Triple X syndrome has been occasionally observed but not systematically investigated so far [61]. Interestingly, essential tremor has been associated with altered cerebellar connectivity (for a review, see 62). So far, these are hypotheses, which is why future studies are needed to systematically investigate the proposed link between structural changes in the cerebellum and motor function in women with 47,XXX.

The traditional view that the cerebellum is involved exclusively in movement control and fine motor skills was debunked more than 50 years ago [63]. More recent studies show that the cerebellum is involved in social-cognitive and especially theory-of-mind functions in addition to other cognitive functions [64]. The ability to attribute mental states to other people is impaired, for example, when the cerebellar structure or function is impaired. These impairments are particularly pronounced if cerebellar development is disturbed in early childhood [65]. Symptoms of the autistic spectrum, for example, can be observed as a direct result of cerebellar damage in early childhood [66]. We therefore hypothesize that the regional volume reduction in the cerebellum seen in the present study could at least be a correlate of the partially autism-like social-cognitive and behavioral problems in individuals with 47,XXX karyotype [9, 10]. However, again, more systematic research involving in-depth exploration of the socio-affective and other cognitive functions is needed. In addition, it should be noted that the cerebellum is associated with various functions, so that the functional significance of the structural changes in the context of this study is not clear [67]. Systematic investigations of structural and functional changes in the cerebellum in the context of different cognitive and motor tasks, as already conducted in autism [64], would be desirable to obtain more specific findings in 47,XXX.

In mammals, one of the two X chromosomes in the female sex, if present, is obligatorily inacti-vated. The heterochromatic chromosome is microscopically visible as the so-called “Barr body” in the nucleus. Inactivation is also obligatory in X chromosome aneuploidy syndromes. X-inactivation is facilitated by the non-coding RNA ‘X inactive-specific transcript’, *XIST* (for review, see 68). However, some genes, such as *RNF12* and *ABCB7*, escape this process, and are regularly expressed from both alleles on the X chromosome to a certain extent. For example, the protein encoded by the murine *Rnf12* gene, ubiquitin protein ligase Rnf12, regulates all transcription factor-encoding genes in the LIM homeodomain family [69, 70], such as *Lmx1b*, which is involved in early cerebellar development [71]. Double-mutant *Lmx1b/Lmx1a* mice do not develop a cerebellum, a condition associated with ataxia [72]. Other members of the LIM homeodomain gene family are involved in many developmental processes affecting brain regions, among these the hippocampus (for review, see 73). The second gene mentioned above, *Abcb7*, is causative for X-linked sideroblastic anemia with ataxia in mice [74]. Given the movement impairments (‘clumsiness’) frequently observed in Triple X syndrome (for review, see 3), processes associated with dysregulated X inactivation and X-linked genes might be involved in impaired development of the cerebellum and other brain areas in Triple X syndrome described here, and require more investigation. Serrarens and colleagues [75] recently demonstrated a a supernumerary X chromosome effect in adult-aged women on intracortical myelin across supragranular laminae of the banks of the superior temporal sulcus and inferior temporal gyrus, both involved in language processing [76] and social cognition [77]. These white matter structural alterations may lead to altered signal transduction in these brain areas, and also be associated with the speech delay [78] and social cognitive impairments [7] observed in gonosomal trisomies.

The present study has some limitations to be considered when interpreting our results. Firstly, our sample of 20 women studied is relatively small, but this corresponds to the usual sample sizes in previous studies. Nonetheless, our main results are statistically significant even after the most conservative correction for multiple testing. Secondly, it must be taken into account that, as in all published studies to date, we only conducted a group comparison and not a longitudinal analysis. Strictly speaking, it is therefore not possible to draw conclusions about the significance of these findings in terms of how the structural changes described here develop; these would have to be investigated in similar studies with a dedicated longitudinal design. Thirdly, all interpretations must consider that the 47,XXX clinical picture is very heterogeneous. While a few women complain of major problems in everyday (social) life, many others are significantly less affected, or not affected at all. Therefore, we cannot guarantee that the sample examined here is representative of the entire population. Finally, it is important to note that our voxel-based analyses results were significantly more robust than the surface-based analyses. The results reported on cortical thickness must be regarded as exploratory and preliminary, and are thus not interpreted here. Studies with larger samples, ideally multicenter studies, would be desirable to investigate regional changes in intracortical structure (e.g. cortical thickness, folding, complexity).

## Conclusions

In sum, this is the first study using VBM and SBM on 3T structural scans in a sample of adult women with 47,XXX that showed marked regional gray matter volume reductions in limbic areas (in particular amygdala and hippocampus), basal ganglia and other subcortical regions, parts of neocortex and, for the first time, in the cerebellum. These alterations in a distributed network of brain regions involved in social cognition and other cognitive functions might thus help us better understand the neural underpinnings of the behavioral characteristics in 47,XXX.

## Electronic supplementary material

Below is the link to the electronic supplementary material.

Supplementary Material 1

## Data Availability

The dataset used and analysed for the current study is available from the corresponding author on reasonable request.
