# Supplementary material for: Brain structure in triple X syndrome: regional gray matter volume and cortical thickness in adult women with 47,XXX karyotype
Source: J Neurodev Disord. 2025 Apr 1;17:18. doi: 10.1186/s11689-025-09608-6 (PMC11959852; doi:10.1186/s11689-025-09608-6)
Supplement: Supplementary file 1 — Supplementary Material 1 [file 11689_2025_9608_MOESM1_ESM.docx]

**Supplementary Materials**

**Brain structure in Triple X syndrome: Regional gray matter volume and cortical thickness in adult women with 47,XXX karyotype**

Gregor Domes^1,2^, Marie-Anne Croyé^3^, Petra Freilinger^4^, Andreas Bohlscheid^5^, Winfried A. Willinek^5^, Jobst Meyer^2,3^

^1^ Department of Biological and Clinical Psychology, University of Trier, Trier, Germany

^2^ Institute for Cognitive and Affective Neuroscience, University of Trier, Trier, Germany

^3^ Department of Neurobehavioral Genetics, University of Trier, Trier, Germany

^4^ Genetikum, Neu-Ulm, Germany

^5^ Department of Radiology, Neuroradiology, Sonography and Nuclear Medicine, Hospital of the Barmherzige Brüder Trier, Trier, Germany

**Table S1**: Structural brain imaging studies involving females with 47,XXX in chronological order

| Study | N | Age M (SD) | Scanner | Analysis | ROIs tested/ Outcomes | Results | Comment |
| --- | --- | --- | --- | --- | --- | --- | --- |
| (Patwardhan et al., 2002) | 10 | 29.1 (2.3) | 1.5 GE Signa | Manual | Amygdala  Hippocampus | ⭘  ⭘ | Amygdala effect at p=0.06 |
| (Lenroot et al., 2014) | 35 | 11.4 (5.5) | NA | CIVET | TBV  Frontal GM  Temporal GM  Parietal GM  Lateral ventricles | 🡫  ⭘  ⭘  🡫  🡩 | WM reductions in all cortical brain regions |
| (Raznahan et al., 2016) | 28^a^ | 12.3 (5.7) | 1.5 GE Signa | CIVET | TBV  Cortical thickness  Cortical surface area | 🡫  🡫🡩  🡫🡩 | Increasing number of X chromosomes associated with alterations in CT & CSA |
| (Reardon et al., 2016) | 23^a^ | 13.0 (6.0) | 1.5 GE Signa | CIVET | Striatum  Pallidum  Thalamus | ⭘  🡫  ⭘ | Effects significant after controlling for TBV |
| (Fish et al., 2017) | 24^a^ | 12.1 (5.4) | 1.5 GE Signa | BrainVISA | Cortical folding  Sulcal length and depth | ⭘  ⭘ | No effects when controlling for TBV |
| (Nadig et al., 2018) | 28^a^ | 12.3 (5.7) | 1.5 GE Signa | CIVET | TBV  Amygdala  Hippocampus | 🡫  🡫  🡫 |  |
| (Serrarens et al., 2022) | 21 | 30.1 (11.8) | 7 T Siemens | Freesurfer; automatic parcellation | ICV  Amygdala  Hippocampus  Putamen  Caudate  Pallidum  Ventral Striate  Thalamus  Lateral ventricles | ⭘  ⭘  🡫  🡫  🡫  🡫  🡫  🡫  🡩 | Not all regions affected bilaterally |

Note. ICV: Intracranial volume; TBV: Total brain volume; CT: Cortical thickness; CSA: Cortical surface area; CV: Cortical volume; CIVET: <https://www.bic.mni.mcgill.ca/ServicesSoftware/CIVET>; BrainVISA: <https://brainvisa.info/web/index.html>

^a^ Study 3-6 investigated a largely overlapping sample

**Table S2.** Educational qualifications of the 47,XXX and control group; distribution by number and percentage

|  | Basic secondary education^1^ | Intermediate secondary education^2^ | Advanced secondary education^3^ | University degree |
| --- | --- | --- | --- | --- |
| 47,XXX | 5 (25.0 %) | 6 (30.0 %) | 6 (30.0 %) | 3 (15.0 %) |
| Controls | 4 (17.4 %) | 9 (39.1 %) | 7 (30.4 %) | 3 (13.0 %) |

Notes. ^1^ equivalent to the German “Hauptschulabschluss” or ”Volksschulabschluss”; ^2^equivalent to the German “Mittlere Reife”; ^3^ equivalent to the German “Abitur” or “Fachhochschulreife”

**Table S3.** VBM GLM results for the contrast Controls > 47,XXX. Significant clusters with decreased GM volume in 47,XXX at a voxel threshold of p<.05 (FWE-corr.); no cluster extent threshold applied

| Region^1^ | Size |  | MNI coordinates | | |  | Peak voxel | | |
| --- | --- | --- | --- | --- | --- | --- | --- | --- | --- |
|  | mm^3^ |  | x | y | Z |  | T | Z | p |
| Hippocampus L | 3851 |  | -29 | -6 | -20 |  | 11.29 | 7.43 | .00000007 |
| Amygdala L |  |  | -26 | -3 | -30 |  | 10.72 | 7.23 | .00000033 |
| Insula L |  |  | -41 | 11 | -9 |  | 8.70 | 6.41 | .00005541 |
| Cerebellum 8 R | 4991 |  | 14 | -72 | -41 |  | 10.43 | 7.12 | .00000070 |
| Cerebellum Crus2 R |  |  | 9 | -78 | -35 |  | 9.40 | 6.72 | .00000917 |
| Cerebellum Crus2 L |  |  | -8 | -75 | -33 |  | 9.35 | 6.70 | .00001023 |
| Amygdala R | 892 |  | 27 | -6 | -15 |  | 9.48 | 6.75 | .00000741 |
| Amygdala R |  |  | 29 | -3 | -24 |  | 8.85 | 6.48 | .00003713 |
| Putamen R | 1680 |  | 35 | 0 | -3 |  | 8.73 | 6.43 | .00005028 |
| Putamen R |  |  | 24 | 6 | -8 |  | 8.25 | 6.21 | .00017707 |
| Insula R |  |  | 38 | 17 | -11 |  | 7.59 | 5.89 | .00102743 |
| Cerebellum 4 5 L | 112 |  | -15 | -33 | -20 |  | 8.41 | 6.28 | .00011584 |
| Cerebellum 4 5 L |  |  | -11 | -38 | -14 |  | 7.34 | 5.76 | .00200160 |
| Cerebellum 9 L | 112 |  | -14 | -44 | -54 |  | 7.81 | 5.99 | .00057612 |
| Cerebellum 9 L |  |  | -15 | -50 | -47 |  | 6.27 | 5.17 | .03485522 |
| OFC ant R | 109 |  | 30 | 48 | -20 |  | 7.75 | 5.96 | .00067992 |
| Vermis 10 | 42 |  | 5 | -41 | -39 |  | 7.67 | 5.93 | .00082500 |
| Cerebellum 9 R | 299 |  | 5 | -48 | -65 |  | 7.65 | 5.92 | .00087472 |
| Cerebellum 9 R |  |  | 9 | -53 | -48 |  | 7.39 | 5.78 | .00174805 |
| Cerebellum 9 R |  |  | 3 | -54 | -71 |  | 7.09 | 5.62 | .00399479 |
| OFC post L | 235 |  | -23 | 14 | -23 |  | 7.57 | 5.87 | .00110046 |
| OFC post L |  |  | -20 | 23 | -20 |  | 7.43 | 5.80 | .00158340 |
| OFC med L |  |  | -15 | 20 | -27 |  | 7.01 | 5.58 | .00491704 |
| Angular L | 10 |  | -48 | -69 | 27 |  | 7.44 | 5.81 | .00153095 |
| OFC post R | 192 |  | 26 | 27 | -14 |  | 7.44 | 5.81 | .00154603 |
| OFC med R |  |  | 18 | 23 | -20 |  | 7.16 | 5.66 | .00327685 |
| OFC med R |  |  | 17 | 23 | -29 |  | 7.08 | 5.62 | .00402730 |
| Cerebellum 4 5 R | 29 |  | 18 | -39 | -14 |  | 7.23 | 5.70 | .00272413 |
| Olfactory R | 74 |  | 3 | 11 | -18 |  | 7.11 | 5.63 | .00375599 |
| Rectus L |  |  | -5 | 14 | -26 |  | 6.30 | 5.18 | .03214069 |
| OFC ant R | 38 |  | 35 | 36 | -14 |  | 6.82 | 5.48 | .00813363 |
| Putamen R | 5 |  | 30 | -5 | 15 |  | 6.72 | 5.42 | .01077889 |
| Vermis 6 | 34 |  | 2 | -59 | -20 |  | 6.69 | 5.41 | .01143050 |
| Vermis 4 5 | 18 |  | 6 | -56 | -12 |  | 6.58 | 5.35 | .01528066 |
| Temporal Sup R | 7 |  | 45 | -20 | -3 |  | 6.49 | 5.29 | .01972271 |
| Parahippocampal L | 6 |  | -15 | 6 | -23 |  | 6.39 | 5.23 | .02570057 |
| Fusiform R | 7 |  | 24 | -53 | -12 |  | 6.37 | 5.22 | .02684124 |
| Cerebellum 8 L | 14 |  | -20 | -66 | -45 |  | 6.31 | 5.19 | .03189520 |
| Temporal Inf L | 5 |  | -27 | -14 | -48 |  | 6.29 | 5.18 | .03308109 |
| Cerebellum 8 L | 5 |  | -21 | -56 | -56 |  | 6.28 | 5.17 | .03395373 |
| Frontal Inf Orb 2 R | 1 |  | 21 | 35 | -5 |  | 6.26 | 5.16 | .03618884 |
| Fusiform L | 5 |  | -33 | -5 | -42 |  | 6.26 | 5.15 | .03657964 |
| Caudate R | 1 |  | 21 | 26 | -8 |  | 6.22 | 5.13 | .04003530 |
| Insula L | 4 |  | -33 | -8 | 12 |  | 6.22 | 5.13 | .04031225 |
| Cerebellum 9 L | 3 |  | -9 | -51 | -57 |  | 6.20 | 5.12 | .04262440 |
| Fusiform L | 1 |  | -20 | -41 | -15 |  | 6.14 | 5.09 | .04935569 |

Notes. ^1^ Peaks are given for cluster peak voxels and local maxima within the clusters

**Table S4.** Partial correlations between autistic traits (AQ), empathy (IRI), anxiety (STAI-T), and depression (BDI) and regional GM volume in selected ROIs in women with 47,XXX and controls; asterisks indicate significant Pearson’s correlations (uncorrected), controlled for age and TIV

|  | AQ | IRI | STAI | BDI |
| --- | --- | --- | --- | --- |
| 1. 47,XXX |  |  |  |  |
| Amygdala L | -.10 | .44 | **-.53*** | -.45 |
| Amygdala R | -.03 | .44 | **-.55*** | **-.54*** |
| Hippocampus L | .02 | .30 | -.36 | -.40 |
| Hippocampus R | -.01 | .30 | **-.49*** | -.42 |
| Putamen L | .25 | .31 | .20 | .18 |
| Putamen R | .19 | .29 | .19 | .21 |
| Cerebellum L | .05 | .14 | -.21 | -.03 |
| Cerebellum R | .09 | .11 | -.31 | -.07 |
| 1. Controls |  |  |  |  |
| Amygdala L | .19 | .06 | .03 | -.01 |
| Amygdala R | .25 | -.03 | .22 | .09 |
| Hippocampus L | -.01 | .15 | -.17 | -.11 |
| Hippocampus R | -.04 | .12 | -.08 | -.04 |
| Putamen L | .05 | -.02 | .25 | .21 |
| Putamen R | .09 | -.05 | .27 | .17 |
| Cerebellum L | -.37 | .24 | -.29 | -.07 |
| Cerebellum R | -.38 | .29 | -.21 | -.07 |

Notes. Df
